# The structure of the first representative of Pfam family PF09836 reveals a two-domain organization and suggests involvement in transcriptional regulation

**DOI:** 10.1107/S1744309109022672

**Published:** 2009-10-27

**Authors:** Debanu Das, Nick V. Grishin, Abhinav Kumar, Dennis Carlton, Constantina Bakolitsa, Mitchell D. Miller, Polat Abdubek, Tamara Astakhova, Herbert L. Axelrod, Prasad Burra, Connie Chen, Hsiu-Ju Chiu, Michelle Chiu, Thomas Clayton, Marc C. Deller, Lian Duan, Kyle Ellrott, Dustin Ernst, Carol L. Farr, Julie Feuerhelm, Anna Grzechnik, Slawomir K. Grzechnik, Joanna C. Grant, Gye Won Han, Lukasz Jaroszewski, Kevin K. Jin, Hope A. Johnson, Heath E. Klock, Mark W. Knuth, Piotr Kozbial, S. Sri Krishna, David Marciano, Daniel McMullan, Andrew T. Morse, Edward Nigoghossian, Amanda Nopakun, Linda Okach, Silvya Oommachen, Jessica Paulsen, Christina Puckett, Ron Reyes, Christopher L. Rife, Natasha Sefcovic, Henry J. Tien, Christine B. Trame, Henry van den Bedem, Dana Weekes, Tiffany Wooten, Qingping Xu, Keith O. Hodgson, John Wooley, Marc-André Elsliger, Ashley M. Deacon, Adam Godzik, Scott A. Lesley, Ian A. Wilson

**Affiliations:** a Joint Center for Structural Genomics, http://www.jcsg.org, USA; b Stanford Synchrotron Radiation Lightsource, SLAC National Accelerator Laboratory, Menlo Park, CA, USA; c The University of Texas Southwestern Medical Center at Dallas, Dallas, TX, USA; d Howard Hughes Medical Institute, Dallas, TX, USA; e Department of Molecular Biology, The Scripps Research Institute, La Jolla, CA, USA; f Program on Bioinformatics and Systems Biology, Burnham Institute for Medical Research, La Jolla, CA, USA; g Protein Sciences Department, Genomics Institute of the Novartis Research Foundation, San Diego, CA, USA; h Center for Research in Biological Systems, University of California, San Diego, La Jolla, CA, USA; i Photon Science, SLAC National Accelerator Laboratory, Menlo Park, CA, USA

**Keywords:** NGO1945, PF09836, DUF2063, putative DNA-binding proteins, putative transcription regulators, structural genomics

## Abstract

Proteins with the DUF2063 domain constitute a new Pfam family, PF09836. The crystal structure of a member of this family, NGO1945 from *Neisseria gonorrhoeae*, has been determined and reveals that the N-terminal DUF2063 domain is likely to be a DNA-binding domain. In conjunction with the rest of the protein, NGO1945 is likely to be involved in transcriptional regulation, which is consistent with genomic neighborhood analysis. Of the 216 currently known proteins that contain a DUF2063 domain, the most significant sequence homologs of NGO1945 (∼40–99% sequence identity) are from various *Neisseria* and *Haemophilus* species. As these are important human pathogens, NGO1945 represents an interesting candidate for further exploration *via* biochemical studies and possible therapeutic intervention.

## Abstract 2

The crystal structure of the NGO1945 gene product from *N. gonorrhoeae* (UniProt Q5F5IO) reveals that the N-terminal domain assigned as a domain of unknown function (DUF2063) is likely to bind DNA and that the protein may be involved in transcriptional regulation.

## 1. Introduction

NGO1945 from *Neisseria gonorrhoeae* FA 1090 is a protein of un­known function with a molecular weight of 28.6 kDa (residues 1–­248) and a calculated isoelectric point of 4.65. It contains an N-­terminal DUF2063 domain (residues 10–92) that has recently been classified as a new Pfam family, PF09836 (Finn *et al.*, 2008 ▶), which is currently comprised of 216 proteins from 173 species (170 from proteobacteria and one each from acidobacteria, bacteroidetes and planctomycetes). Pfam also indicates that this domain is observed in 68 sequences from NCBI’s METASEQ metagenomics data set. Of these 216 proteins, 215 have a single N-terminal DUF2063 domain and, in one instance, this domain is found with a DUF692 domain (PF05114; TIM barrel superfamily, Pfam clan CL0152, which includes endonuclease IV), in which the DUF692 domain is at the N-terminus. Bioinformatics analysis (Altschul *et al.*, 1997 ▶; Marchler-Bauer *et al.*, 2007 ▶; Jaroszewski *et al.*, 2005 ▶; Tatusov *et al.*, 2000 ▶) revealed that of the significant sequence homologs of NGO1945, five proteins are from different species of *Neisseria* (94–99% sequence identity) and 14 (including predicted RNA polymerase sigma factor and repair proteins) are from different strains of *Haemophilus* (40–90% sequence identity). We have determined the crystal structure of NGO1945 to 2.1 Å resolution in order to expand structural coverage of novel protein-sequence space and assign putative functions to novel proteins that have not been previously studied and whose functions cannot be inferred from sequence homology. This study allows us to assign a putative molecular function to the NGO1945 protein and to the DUF2063 domain.

## 2. Materials and methods

### 2.1. Protein production and crystallization

Clones were generated using the Polymerase Incomplete Primer Extension (PIPE) cloning method (Klock *et al.*, 2008 ▶). The gene encoding NGO1945 (GenBank YP_208969) was amplified by polymerase chain reaction (PCR) from *N. gonorrhoeae* FA 1090 genomic DNA using *PfuTurbo* DNA polymerase (Stratagene) and I-PIPE primers (forward primer, 5′-ctgtacttccagggcATGCAGCCTGAAAC­CTCCGCCCAATACC-3′; reverse primer, 5′-aattaagtcgcgttaTGCGGATAGATGGTTTTGGCTCGGGG-3′; target sequence in upper case) that included sequences for the predicted 5′ and 3′ ends. The genomic DNA used here contained a single amino-acid substitution, P94L, when compared with the available GenBank sequence from *N. gonorrhoeae* FA 1090. The expression vector pSpeedET, which encodes an amino-terminal tobacco etch virus (TEV) protease-cleavable expression and purification tag (MGSDKIHHHHHHENLYFQ/G), was PCR-amplified with V-PIPE primers (forward primer, 5′-taacgcgacttaattaactcgtttaaacggtctccagc-3′; reverse primer, 5′-gcc­ctggaagtacaggttttcgtgatgatgatgatgatg-3′). The V-PIPE and I-PIPE PCR products were mixed to anneal the amplified DNA fragments together. *Escherichia coli* GeneHogs (Invitrogen) competent cells were transformed with the V-PIPE/I-PIPE mixture and dispensed onto selective LB–agar plates. The cloning junctions were confirmed by DNA sequencing. Expression was performed in selenomethionine-containing medium. At the end of fermentation, lysozyme was added to the culture to a final concentration of 250 µg ml^−1^ and the cells were harvested and frozen. After one freeze–thaw cycle, the cells were homogenized in lysis buffer [50 m*M* HEPES pH 8.0, 50 m*M* NaCl, 10 m*M* imidazole, 1 m*M* tris(2-carboxyethyl)phos­phine–HCl (TCEP)] and the lysate was clarified by centrifugation at 32 500*g* for 30 min. The soluble fraction was passed over nickel-chelating resin (GE Healthcare) pre-equilibrated with lysis buffer, the resin was washed with wash buffer [50 m*M* HEPES pH 8.0, 300 m*M* NaCl, 40 m*M* imidazole, 10%(*v*/*v*) glycerol, 1 m*M* TCEP] and the protein was eluted with elution buffer [20 m*M* HEPES pH 8.0, 300 m*M* imidazole, 10%(*v*/*v*) glycerol, 1 m*M* TCEP]. The eluate was buffer-exchanged with HEPES crystallization buffer (20 m*M* HEPES pH 8.0, 200 m*M* NaCl, 40 m*M* imidazole, 1 m*M* TCEP) using a PD-10 column (GE Healthcare) and incubated with 1 mg TEV protease per 15 mg eluted protein. The protease-treated eluate was passed over nickel-chelating resin (GE Healthcare) pre-equilibrated with HEPES crystallization buffer and the resin was washed with the same buffer. The flowthrough and wash fractions were combined and concentrated to 16.2 mg ml^−1^ by centrifugal ultrafiltration (Millipore) for crystallization trials. NGO1945 was crystallized using the nanodroplet vapor-diffusion method (Santarsiero *et al.*, 2002 ▶) with standard JCSG crystallization protocols (Lesley *et al.*, 2002 ▶) by mixing 100 nl protein solution with 100 nl crystallization solution and equilibrating against a 50 µl reservoir volume. The crystallization reagent contained 0.2 *M* magnesium chloride, 8.2%(*v*/*v*) ethanol and 0.1 *M* imidazole pH 8.36. A rod-shaped crystal of approximate dimensions 20 × 20 × 100 µm was harvested after 34 d at 277 K for data collection. To determine its oligomeric state in solution, NGO1945 was analyzed using a 1 × 30 cm Superdex 200 size-exclusion column (GE Healthcare) coupled with miniDAWN static light-scattering (SEC/SLS) and Optilab differential refractive-index detectors (Wyatt Technology). The mobile phase consisted of 20 m*M* Tris pH 8.0, 150 m*M* NaCl and 0.02%(*w*/*v*) sodium azide. The molecular weight was calculated using *ASTRA* 5.1.5 software (Wyatt Technology).

### 2.2. Data collection, structure solution and refinement

No additional cryoprotectant was added to the crystal during data collection. Initial screening for diffraction was carried out using the Stanford Automated Mounting system (SAM; Cohen *et al.*, 2002 ▶) at the Stanford Synchrotron Radiation Lightsource (SSRL; Menlo Park, California, USA). The diffraction data were indexed in the monoclinic space group *C*2. Multi-wavelength anomalous diffraction (MAD) data were collected at SSRL on beamline BL9-2 at wavelengths corresponding to the high-energy remote (λ_1_) and inflection (λ_2_) of a selenium MAD experiment. The data sets were collected at 100 K using a MarMosaic 325 CCD detector. The MAD data were integrated and reduced using *MOSFLM* (Leslie, 1992 ▶) and scaled with the program *SCALA* from the *CCP*4 suite (Collaborative Computational Project, Number 4, 1994 ▶). Phasing was performed with *SOLVE* (Terwilliger & Berendzen, 1999 ▶) and automated model building was performed with *ARP*/*wARP* (Perrakis *et al.*, 1999 ▶) and *RESOLVE* (Terwilliger, 2000 ▶). Model completion was performed with *Coot* (Emsley & Cowtan, 2004 ▶). Refinement was performed with *REFMAC*5 (Winn *et al.*, 2003 ▶) with one TLS group, using the high-energy remote (λ_1_) data set and excluding diffraction maxima present in ice rings spanning the resolution ranges 3.97–3.82, 3.72–3.61, 3.50–3.39, 2.69–2.64 and 2.26–2.24 Å and an additional 14 unusually strong reflections (near ice rings) that had intensities 15× greater than the average intensity for their resolution bin. The removal of reflections affected by ice rings resulted in a difference in completeness between the λ_1_ and λ_2_ data sets. To ensure that the 14 unusually strong reflections were indeed spurious (near ice rings) and did not reflect real intensities from the protein component of the crystal, we calculated structure factors from the final model after refinement, which revealed that these 14 reflections were not unusually strong. This analysis confirmed that the 14 reflections in the measured data should be removed from the refinement data set. Crystallographic data and refinement statistics are summarized in Table 1 ▶.

### 2.3. Validation and deposition

**Figure 1:**
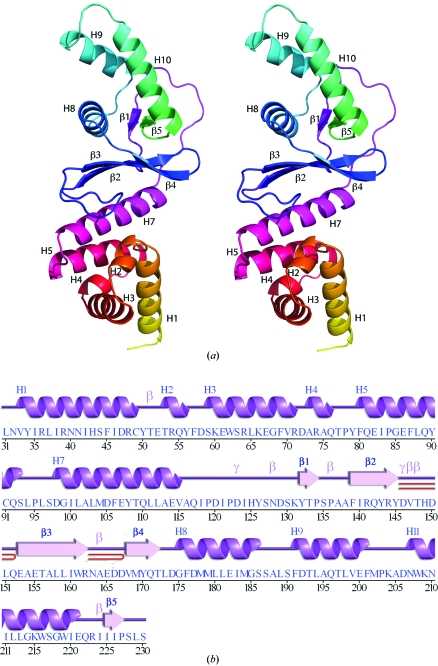
Crystal structure of NGO1945 from *N. gonorrhoeae* FA 1090. (*a*) Stereo ribbon diagram of the NGO1945 monomer color coded from the N-terminus (yellow) to the C-­terminus (green). Helices H1–H11 and β-strands β1–β5. (*b*) Diagram showing the secondary-structure elements of NGO1945 superimposed on its primary sequence. The labeling of secondary-structure elements is in accord with *PDBsum* (http://www.ebi.ac.uk/pdbsum), where α-helices are sequentially labeled (H1–H11), β-strands are labeled (β1–β5), β-turns and γ-turns are designated by Greek letters (β, γ) and β-­hairpins by red loops.

The quality of the crystal structure was analyzed using the JCSG Quality Control server, which verifies the stereochemical quality of the model using *AutoDepInputTool* (Yang *et al.*, 2004 ▶), *MolProbity* (Davis *et al.*, 2004 ▶) and *WHATIF 5.0* (Vriend, 1990 ▶), the agreement between the atomic model and the data using *SFCHECK* 4.0 (Vaguine *et al.*, 1999 ▶) and *RESOLVE* (Terwilliger, 2000 ▶), the protein sequence using *ClustalW* (Thompson *et al.*, 1994 ▶) and the atom occupancies using *MOLEMAN*2 (Kleywegt, 2000 ▶). It also evaluates the difference in *R*_cryst_/*R*_free_, expected *R*_free_/*R*_cryst_ and maximum/minimum *B* values by parsing the refinement log file and PDB header. Protein quaternary-structure analysis was performed using the *PISA* server (Krissinel & Henrick, 2005 ▶). Fig. 1 ▶(*b*) was adapted from an analysis using *PDBsum* (Laskowski *et al.*, 2005 ▶) and all other figures were prepared with *PyMOL* (DeLano, 2002 ▶). Atomic co­ordinates and experimental structure factors have been deposited in the PDB under accession code 3dee.

## 3. Results and discussion

### 3.1. Overall structure

**Figure 2:**
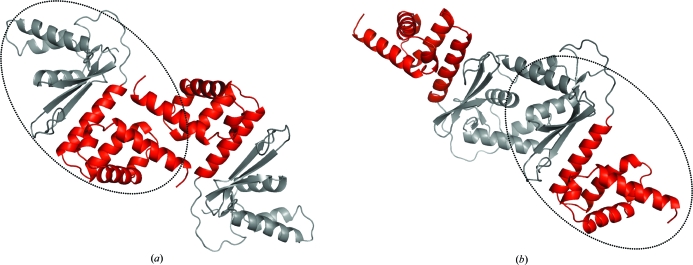
NGO1945 dimerization. Based on crystal-packing analysis, dimerization may occur *via* (*a*) N-terminal domain helices H1, H5 and H7, with a total buried surface area of 1550 Å^2^ (red), or (*b*) C-terminal domain helices H8, H9 and H11, with a total buried surface area of 1930  Å^2^ (gray) (for clarity, the monomer encircled by a dashed oval is depicted in approximately the same orientation in both panels).

The crystal structure of NGO1945 was determined to 2.10 Å resolution using the MAD method (Fig. 1 ▶). Data-collection, model and refinement statistics are summarized in Table 1 ▶. The final model includes one monomer (residues 31–230), two chloride ions, one imidazole molecule and 95 water molecules in the asymmetric unit. No electron density was observed for residues 1–30 and 231–248, even though LC-MS confirmed their presence in the purified protein before crystallization. Thus, they are either disordered in the crystal (note that there is sufficient space in the lattice to accommodate the disordered residues) or the protein may have undergone limited proteolysis in the crystallization drop. The Matthews coefficient (*V*_M_; Matthews, 1968 ▶) is ∼2.6 Å^3^ Da^−1^ and the estimated solvent content is ∼53%. The Ramachandran plot produced by *MolProbity* (Davis *et al.*, 2004 ▶) shows that 96.5% of the residues are in favored regions, with no outliers. Crystal-packing analysis predicts that NGO1945 may dimerize *via* its N-terminal or C-terminal domains [using helices H1, H5 and H7 (Fig. 2 ▶ *a*) or helices H8, H9 and H11 (Fig. 2 ▶ *b*), respectively] with total buried surface areas of 1550 and 1930 Å^2^ and Δ*G* ^int^ values of −53.2 and −82.0 kJ mol^−1^, respectively. Analytical size-exclusion chromatography in combination with static light scattering (SEC/SLS) revealed the oligomeric form in solution to be a dimer, but with medium confidence (∼30% was clearly dimeric, ∼50% was interchanging between monomer and dimer and ∼20% was likely to be monomeric, suggesting that at any given time the dimer:monomer ratio was ∼60:40). An imidazole molecule (likely to be from the buffer) is bound on the surface to Glu114, Asp166 and Arg162, but its significance is known.

A systematic search for other proteins of similar structure was conducted using several different methods, including the *DALI* server (Holm *et al.*, 2008 ▶), the protein structure-comparison service *SSM* at the European Bioinformatics Institute (http://www.ebi.ac.uk/msd-srv/ssm; Krissinel & Henrick, 2005 ▶) and the flexible structure-alignment method *FATCAT* (Ye & Godzik, 2004 ▶). No significant matches of the full protein structure with any other known protein structures were found. However, significant structural similarities are seen with other proteins when queried with individual NGO1945 domains, as discussed below.

### 3.2. N-terminal domain

**Figure 3:**
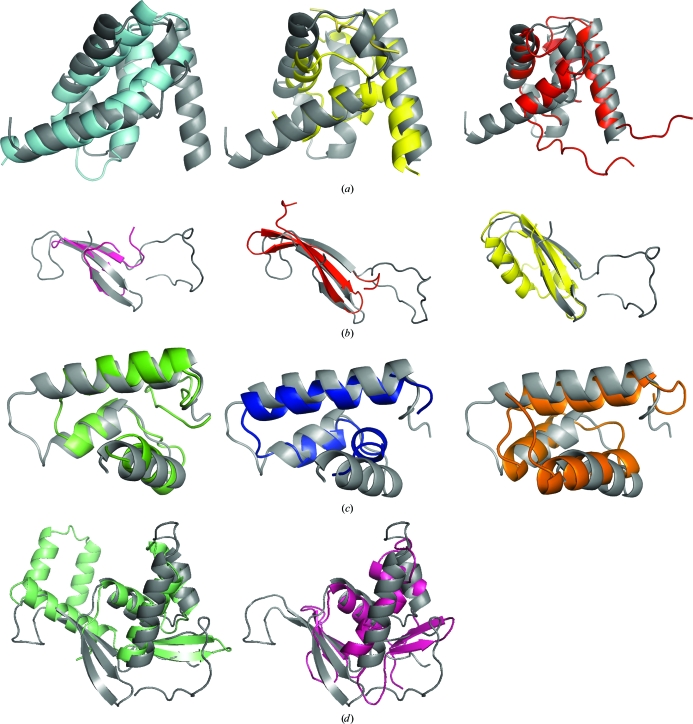
Domain structure comparisons. (*a*) Superimposition of the DUF2063 domain (residues 31–116) of NGO1945 (gray) with the σ2 domain of RNA polymerase sigma factor SigR from *S. coelicolor* (PDB code 1h3l, cyan), the SAM domain of the transcription elongation factor NusA (1u9l, yellow) and the N-terminal domain of STE50 (1uqv, red). (*b*) Superimposition of NGO1945 residues 117–174 (gray) with the WW domain of human FE65 (2idh, pink), monellin (3mon, red) and the N-terminal domain of the ribosomal protein L11 from *T. maritima* (1mms, yellow). (*c*) Comparison of NGO1945 residues 175–231 (gray) with the Z-DNA-binding domain of the vaccinia virus E3L protein (1oyi, green), the DNA-binding domain of MafG bZIP (1k1v, blue) and the Nanog homeodomain (2vi6, orange). (*d*) Superimposition of NGO1945 residues 117–231 (gray) with the transcription repressor MecI (1okr, green) and the DNA-binding domain of the response regulator PhoP (2pmu, pink).

**Figure 4:**
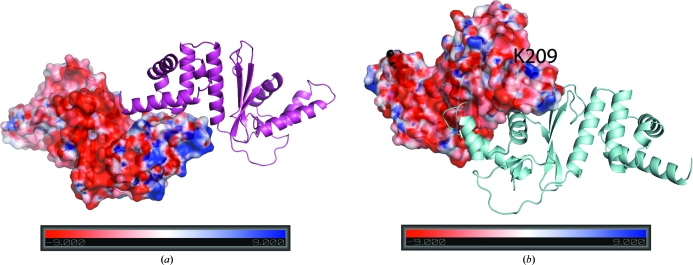
Electrostatic surface of potential NGO1945 dimers. (*a*) The N-terminal dimer. The monomer on the left is drawn as an electrostatic surface (± *kT*/e), which highlights the basic patch (Arg36, Arg39, Arg48, Arg54, Lys60, Arg64, Lys66, Arg71 and Arg74) that may be involved in DNA/RNA-binding interactions. This basic region is distinct from the portion involved in the protein–protein interactions that form the dimer. These residues are conserved in many proteins belonging to DUF2063 (Pfam website alignment), suggesting functional importance. (*b*) The C-terminal dimer. The monomer on the left is represented as an electrostatic surface, which highlights the surface-exposed Lys209 in NGO1945 corresponding to Lys40 that is implicated in Z-DNA binding in vaccinia virus E3L protein. The other E3L protein residues implicated in Z-DNA binding, Arg41, Asn44, Lys45, Tyr48 and Trp66, are not conserved in NGO1945.

The ordered region of the NGO1945 N-terminal domain (residues 31–116) includes the DUF2063 domain (Pfam PF09836; residues 31–92) and is similar to many proteins that contain α-helical bundles. Some of the significant hits are with the σ2 domain of RNA polymerase sigma factor SigR from *Streptomyces coelicolor* [Li *et al.*, 2002 ▶; Burgess & Anthony, 2001 ▶; PDB code 1h3l; SCOP fold 88945, *DALI Z* score 4.4 (*Z* scores above 2.0 are significant), 2.9 Å r.m.s.d. over 61 C^α^ atoms, 0% sequence identity; Fig. 3 ▶ *a*] and the σ3 domain of sigma factor σ^28^ FliA from *Aquifex aeolicus* (Sorenson *et al.*, 2004 ▶; PDB code 1rp3; SCOP fold 46688, *Z* score 3.9, 3.3 Å r.m.s.d. over 61 C^α^ atoms, 5% sequence identity). The N-terminal domain is also similar to the ∼70-residue SAM (sterile alpha motif) domain (SCOP fold 47768), which contains a helix–hairpin–helix (HhH) motif (Shao & Grishin, 2000 ▶) that is found in several hundred proteins that are involved in signal transduction and transcriptional regulation (Grimshaw *et al.*, 2004 ▶). For example, the SAM domain is found within the C-terminal domain of the transcription elongation factor NusA (Bonin *et al.*, 2004 ▶; PDB code 1u9l; *Z* score 4.2, 2.5 Å r.m.s.d. over 57 C^α^ atoms, 11% sequence identity; Fig. 3 ▶ *a*), which is involved in interaction with the C-terminal domain (CTD) of the α subunit of RNA polymerase to inhibit RNA binding during transcription termination. It is also seen as the N-terminal domain of STE50, a modulator of mitogen-activated protein kinase signaling in yeast (Grimshaw *et al.*, 2004 ▶; PDB code 1uqv; *Z* score 3.6, 2.80 Å r.m.s.d. over 61 C^α^ atoms, 10% sequence identity; Fig. 3 ▶ *a*). SAM domains are involved in protein–protein interactions, either for self-association or for binding to non-SAM-domain proteins (Peterson *et al.*, 1997 ▶). SAM domains are also implicated in RNA binding, as in the case of the positively charged residues in the SAM domain of Smaug (PDB code 1oxj; *Z* score 2.4, 3.10 Å r.m.s.d. over 57 C^α^ atoms with 14% sequence identity to the N-terminal domain of NGO1945; Green *et al.*, 2003 ▶; Kim & Bowie, 2003 ▶). The Smaug SAM domain has been defined as a new family of regulators of post-transcriptional control (Aviv *et al.*, 2003 ▶). However, none of the functionally important residues in any of the proteins discussed above are conserved in NGO1945. Inspection of the putative N-terminal dimer (Fig. 2 ▶ *a*) and electrostatic surfaces (Fig. 4 ▶ *a*) support the possibility of this domain being involved in protein–protein interactions, as well as DNA/RNA binding, *via* charged residues in a basic patch (separate from the dimerization interface) that is comprised of Arg36, Arg39, Arg48, Arg54, Lys60, Arg64, Lys66, Arg71 and Arg74. The surface-exposed aromatic residues Tyr34, Phe45, Trp62 and Phe80 in the monomer, which are mostly hidden in an N-terminal dimer, could play a role in base-stacking interactions with DNA if the functional form of the protein requires dissociation into monomers, but this is only speculation at this point. Phe13 and Arg18 in the disordered N-terminus may also become ordered on binding to DNA and are conserved in many PF09836 proteins (Pfam alignment), suggesting functional importance.

### 3.3. C-terminal domain

An ∼20-residue loop (residues 117–137) connects the N-terminal domain to the remainder of the protein and could facilitate movement of the N-terminal and C-terminal domains with respect to each other. Residues 138–174 (Fig. 3 ▶ *b*) are similar in structure to the WW domain (a three-stranded β-sheet structure, but without the characteristic tryptophan residues) of human FE65 (Meiyappan *et al.*, 2007 ▶; PDB code 2idh; *Z* score 1.7, 1.98 Å r.m.s.d. over 27 C^α^ atoms, 7% sequence identity) and to the intensely sweet protein monellin (Ogata *et al.*, 1987 ▶; PDB code 3mon; *Z* score 1.9, 2.5 Å r.m.s.d. over 32 C^α^ atoms, 3% sequence identity). Some similarity is also found to the N-terminal domain of the ribosomal protein L11 from *Thermotoga maritima* (Wimberly *et al.*, 1999 ▶; PDB code 1mms; *Z* score 1.6, 3.0 Å r.m.s.d. over 36 C^α^ atoms, 11% sequence identity). Thus, this region (residues 138–174) with its negatively charged surface (Fig. 4 ▶ *a*) may be involved in interactions with a binding partner.

Residues 175–231 of NGO1945 are similar to DNA-binding proteins (Fig. 3 ▶ *c*) belonging to SCOP fold 46688, examples of which include the Z-DNA-binding domain of the vaccinia virus E3L protein (Kahmann *et al.*, 2004 ▶; PDB code 1oyi; *Z* score 4.6, 1.76 Å r.m.s.d. over 44 C^α^ atoms, 9% sequence identity) and the globular DNA-binding domain of the histone protein H5 (GH5; Ramakrishnan *et al.*, 1993 ▶; PDB code 1hst; *Z* score 2.0, 3.0 Å r.m.s.d. over 49 C^α^ atoms, 6% sequence identity). Of the E3L residues that are implicated in Z-­DNA binding (Lys40, Arg41, Asn44, Lys45, Tyr48 and Trp66), the only residue that is conserved in NGO1945 is Lys209 (corresponding to Lys40 in E3L; Fig. 4 ▶ *b*). The GH5 residues that are involved in DNA binding include His25, Lys40, Arg42, Lys52, His62, Lys69, Arg73, Lys85 and Arg94, none of which are conserved in NGO1945. Significant similarity also occurs with DNA-binding eukaryotic transcription factors (Fig. 3 ▶ *c*) in SCOP fold 47453 (superfamily 47454), such as the DNA-binding domain of the MafG bZIP transcription factor (Kusunoki *et al.*, 2002 ▶; PDB code 1k1v; *Z* score 2.4, 2.3 Å r.m.s.d. over 37 C^α^ atoms, 16% sequence identity), the Nanog homeodomain transcription factor (Jauch *et al.*, 2008 ▶; PDB code 2vi6; *Z* score 2.7, 2.7 Å r.m.s.d. over 43 C^α^ atoms, 7% sequence identity) and the γ-domain of DNA translocase FtsK (Lowe *et al.*, 2008 ▶; PDB code 2ve8; *Z* score 3.1, 2.23 Å r.m.s.d. over 44 C^α^ atoms, 9% sequence identity). The Nanog residues that could bind DNA are Lys43, Thr47, Gln50, Asn51 and Met54, whereas those in MafG are Lys53, Arg56, Arg57, Lys60, Asn61, Tyr64, Ala65, Cys68 and Arg69. As in E3L, the DNA-binding residues are on the same DNA recognition helix (the top helix in Fig. 3 ▶ *c*). None of these Nanog and MafG residues are conserved in NGO1945.

When residues 117–231 are considered as a single domain, it appears to be a circular permutation of the wHTH (winged helix–turn–helix) motif in which the ‘wing’ precedes the helices, as in the transcription repressor MecI (PDB code 1okr; *Z* score 3.4, 2.7 Å r.m.s.d. over 57 C^α^ atoms, 5% sequence identity; Fig. 3 ▶ *d*). A β-­strand preceding the HTH portion in a wHTH domain is also seen in the DNA-binding domain of the response regulator PhoP (PDB code 2pmu; *Z* score 3.5, 3.4 Å r.m.s.d. over 71 C^α^ atoms, 11% sequence identity; Fig. 3 ▶ *d*). The PhoP residues that may interact with DNA (Wang *et al.*, 2007 ▶) are Lys197, Trp203, Asn212, Val213, Glu215, Ser216, Arg223, Lys224 and Arg237 (similar to the PhoB DNA-binding residues) and the corresponding MecI residues are Lys43, Arg46, Thr47, Thr50, Arg51, Lys54 and Lys55 (Garcia-Castellanos *et al.*, 2003 ▶). However, these residues are not conserved in NGO1945.

### 3.4. Genome-context analysis

Analysis of the *ngo1945* phylogenetic co-occurrence and genomic neighborhood in related species (Jensen *et al.*, 2009 ▶) predicts some other proteins with which it may have functional associations, including NGO1943 (unknown function), NGO1944 ▶ (Pfam PF04542, domain 2 of σ^70^ ECF RNA polymerase sigma factors), NGO1946 (unknown function DUF692; PF05114), NGO1947 (putative periplasmic protein of unknown function; Gunesekere *et al.*, 2006 ▶), NGO1948 (DoxX; PF07681, similar to DoxD, the small subunit of the terminal quinol oxidase; PF04173, potential integral membrane protein involved in sulfur oxidation), NGO1198 (PF00884, sulfatase), NGO2105 (putative adhesion penetration protein) and NGO1482 (unknown function DUF452; PF04301). Functional studies with NGO1944 based on DNA microarrays suggest that NGO1944, NGO1945, NGO1946, NGO1947 and NGO1948 may be cotranscribed and involved in the regulation of *msrAB*, a methionine sulfoxide reductase (Gunesekere *et al.*, 2006 ▶).

The genomic context of other DUF2063 homologs supports an involvement in virulence. For example, MCA3109 and ABO1516, which are DUF2063 homologs from *Methylococcus capsulatus* and *Alcanivorax borkumensis*, respectively, show a predicted functional association with lipoprotein VacJ. The *vacJ* gene is required for intercellular spreading and virulence in *Shigella flexneri* and entero­invasive *E. coli* (Suzuki *et al.*, 1994 ▶). *H. influenzae* homologs, such as NTHI1444 and HI1599, co-occur with hemoglobin–haptoglobin binding proteins that are virulence determinants (Seale *et al.*, 2006 ▶). Similarly, *Pseudomonas aeruginosa* homologs co-occur with heme-exporter protein D (a cytochrome *c*-type biogenesis protein) that has been implicated in invasion and virulence in *Legionella pneumophila* (Polesky *et al.*, 2001 ▶), while *Burkholderia* and *Bordetella* homologs are present with hemolysin-related and exported proteins.

In conclusion, the NGO1945 crystal structure allows the assignment of a putative function for this protein and for the PF09836 family in general. Structural similarity to transcription factors and presence of a surface-exposed basic patch in the N-terminal DUF2063 domain indicates the possibility of DNA binding. The nonconservation of DNA-binding residues in NGO1945 compared with structurally similar proteins may give rise to a different mode of DNA binding. The multi-domain architecture, potential DNA binding and genome context of *ngo1945* are consistent with a possible role in transcription pathways and indicate that the members of this family may be transcription factors; the genome context further supports involvement in virulence. Alternatively, they may have some novel functionality that remains to be determined. Since significant sequence homologs of NGO1945 are primarily found in different strains of the bacteria *Neisseria* and *Haemophilus*, which are human pathogens that are involved in sexually transmitted diseases, in meningitis and in ear, eye or sinus infections in infants and children, further structure-based biochemical investigation of NGO1945 may be of therapeutic value.

Additional information about NGO1945 is available from TOPSAN (Krishna *et al.*, 2010 ▶) http://www.topsan.org/explore?PDBid=3dee.

## Supplementary Material

PDB reference: NGO1945 from *N. gonorrhoeae*, 3dee, r3deesf

## Figures and Tables

**Table 1:** Crystallographic data and refinement statistics for NGO1945 (PDB code 3dee) Values in parentheses are for the highest resolution shell.

	λ_1_ MADSe	λ_2_ MADSe
Space group	*C*2	
Unit-cell parameters (Å, °)	*a* = 106.52, *b* = 31.88, *c* = 86.37, β = 115.80	
Data collection		
Wavelength (Å)	0.9184	0.9793
Resolution range (Å)	77.9–2.10 (2.21–2.10)	50.0–2.10 (2.15–2.10)
No. of observations	41439	45988
No. of unique reflections	13583	15291
Completeness (%)	87.2 (97.9)	98.1 (97.7)
Mean *I*/σ(*I*)	10.9 (2.3)	9.3 (1.7)
*R*_merge_ on *I*† (%)	8.8 (47.5)	10.9 (52.5)
*R*_meas_ on *I*‡ (%)	10.7 (57.3)	13.7 (63.4)
Model and refinement statistics		
Resolution range (Å)	77.9–2.10	
No. of reflections (total)	13555§	
No. of reflections (test)	660	
Completeness (%)	86.5	
Data set used in refinement	λ_1_	
Cutoff criterion	|*F*| > 0	
*R*_cryst_¶	0.223	
*R*_free_††	0.267	
Stereochemical parameters		
Restraints (r.m.s.d. observed)		
Bond angles (°)	1.54	
Bond lengths (Å)	0.015	
Average isotropic *B* value (Å^2^)	37.8‡‡	
ESU§§ based on *R*_free_ (Å)	0.22	
Protein residues/atoms	200/1633	
Waters/ions	95/3	

† *R*_merge_ = 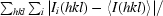 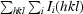.

‡ *R*_meas_ = 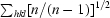 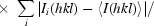 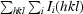 (Diederichs & Karplus, 1997 ▶).

§ Typically, the number of unique reflections used in refinement is slightly less than the total number that were integrated and scaled. Reflections are excluded owing to negative intensities and rounding errors in the resolution limits and unit-cell parameters. In addition, ice-ring regions were excluded prior to integration and 14 reflections with intensity greater than 15 times the average for their shell were omitted prior to refinement.

¶ *R*_cryst_ = 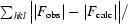 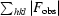, where *F*_calc_ and *F*_obs_ are the calculated and observed structure-factor amplitudes, respectively.

†† *R*_free_ is the same as *R*_cryst_ but for 4.9% of the total reflections chosen at random and omitted from refinement.

‡‡ This value represents the total *B*, which includes TLS and residual *B* components.

§§ Estimated overall coordinate error (Collaborative Computational Project, Number 4, 1994 ▶; Cruickshank, 1999 ▶).
